# Gene therapy of prostate cancer using liposomes containing perforin expression vector driven by the promoter of prostate-specific antigen gene

**DOI:** 10.1038/s41598-021-03324-6

**Published:** 2022-01-27

**Authors:** Kosuke Mizutani, Kyojiro Kawakami, Yasunori Fujita, Taku Kato, Manabu Takai, Daiki Kato, Koji Iinuma, Takuya Koie, Masafumi Ito

**Affiliations:** 1 Department of Urology, Gifu University Graduate School of Medicine, 1-1 Yanagido, Gifu, Gifu, 501-1194 Japan; 2 Department of Urology, Kizawa Memorial Hospital, Minokamo, Gifu, 505-8503 Japan; 3 Research Team for Mechanism of Aging, Tokyo Metropolitan Institute of Gerontology, 35-2 Sakae-cho, Itabashi-ku, Tokyo, 173-0015 Japan; 4 Research Team for Functional Biogerontology, Tokyo Metropolitan Institute of Gerontology, 35-2 Sakae-cho, Itabashi-ku, Tokyo, 173-0015 Japan; 5 Department of Urology, Asahi University Hospital, 3-23 Hashimotocho, Gifu, Gifu 500-8523 Japan

**Keywords:** Cancer immunotherapy, Targeted therapies, Cancer, Prostate cancer

## Abstract

Perforin secreted from cytotoxic lymphocytes plays a critical role in cancer immunosurveillance. The aim of this study was to investigate the therapeutic potential of liposomes containing perforin expression vector driven by the promotor of prostate-specific antigen (PSA). The anti-tumor effect of perforin was analyzed using prostate cancer (PC) PC-3 cells in which perforin expression was controlled by Tet-on system (PC-3PRF cells). Liposomes encapsulating PSA promoter-driven perforin expression vector (pLipo) were constructed for its specific expression in PC. The anti-tumor effect of pLipo was evaluated in vitro using docetaxel-resistant PC 22Rv1 PC cell line, 22Rv1DR, and PC-3 cells in the presence of human peripheral blood mono nuclear cells (PBMCs) and also in vivo using male nude mice bearing 22Rv1DR cell-derived tumor xenograft. Induction of perforin significantly inhibited growth of PC-3PRF cells. Treatment with pLipo induced perforin expression in 22Rv1DR cells expressing PSA but not in PC-3 cells lacking it. Treatment with pLipo at a low concentration was prone to inhibit growth of both cell lines and significantly inhibited growth of 22Rv1DR cells when co-incubated with PBMCs. The combined use of pLipo at a high concentration with PBMCs showed nearly complete inhibition of 22Rv1DR cell growth. Intravenous administration of pLipo via tail vein increased the level of perforin in tumor and serum and significantly decreased the tumor volume. Our results suggest that liposome-mediated PC-specific expression of perforin could be a novel therapy for advanced PC.

## Introduction

Cancer caused death reached nearly 10 million in 2020 in the world^1^. Advanced cancer has been treated mostly by chemotherapy and targeted therapy using small molecule inhibitors. After intensive research on the potential of immunotherapy over the past decade, the first immune checkpoint inhibitor (ICI) was approved in 2011, which targeted cytotoxic lymphocyte antigen 4^2^. ICIs significantly improved overall survival in several types of cancer, however, more than half of the patients are still incurable^3–5^. To improve the prognosis, novel therapies based on the mechanism of immune responses to cancer are urgently needed.

Cyotoxic T lymphocytes (CTLs) and natural killer (NK) cells play pivotal roles in the anti-tumor immune response leading to cancer cell apoptosis^6,7^. Although mechanisms underlying the activation of immune cells may vary, immune synapse is eventually formed, where perforin and granzymes are secreted from CTLs and NK cells: perforin makes pores on the plasma membranes of target cells, through which granzymes enter into the target cell cytoplasm, resulting in DNA cleavage and apoptosis. The function of perforin is critically important in protection of human from infectious diseases and malignancies, therefore, mutations of the perforin gene (*PRF1*) cause hemophagocytic lymphohistiocytosis and induce hematological malignancies^8–10^. Recently, a link between partial loss of perforin function and solid tumors was proposed. In the microenvironment of prostate cancer (PC), perforin expression in CTLs and NK cells was diminished^11^. In colorectal cancer, the number of perforin expressing CD8+ or CD16+ cells was lowered, which correlated with tumor progression^12^. For these reasons, perforin could be an attractive therapeutic target in cancer immunotherapy. On the other hand, perforin-dependent cytotoxicity was suggested in Type 1 juvenile diabetes, cerebral malaria and multiple sclerosis^9,13^, therefore adverse effect of perforin expression in cancer therapy should be fully considered.

PC is the fifth leading mortality cause in men^1^. Although Sipuleucel-T vaccine, an active cellular immunotherapy, improved overall survival among men with metastatic castration-resistant PC (CRPC), therapeutic effects of ICIs are limited for various reasons: low tumor mutation burden with less number of neoantigens, insufficient tumor microenvironment accompanying fewer CD8+ T cells and increased number of regulatory T cells and less PD-L1 expression in advanced PC^14,15^. Previous reports showed that less perforin expression in tumor and serum correlated with progression of lung cancer and that direct injection of perforin expression vector into xenografted tumors reduced their size^16,17^.

In the present study, we aimed to increase the concentration of perforin in the microenvironment of PC in order to enhance the anti-tumor immune response mediated by CTLs and NK cells. Since overexpression of perforin in immune cells in vivo is highly likely to cause systemic adverse events, we expressed it specifically in PC cells by using liposomes containing its expression vector driven by the promoter of prostate-specific antigen (PSA) gene. After confirming the anti-tumor effect of the engineered liposomes in PC cells expressing PSA in the presence of human peripheral blood mononuclear cells (PBMCs), their effect against tumor growth in vivo was analyzed using a mouse xenograft model harboring chemo-resistant CRPC. Intravenous administration of the liposomes increased the level of perforin in tumor and serum and decreased the tumor volume, providing evidence for the therapeutic potential of perforin expression in PC.

## Materials and methods

### Cell lines

Human PC cell lines LNCaP and PC-3 obtained from JCRB Cell Bank (Osaka, Japan), and 22Rv1 purchased from The European Collection of Authenticated Cell Cultures were maintained in RPMI-1640 (FUJIFILM Wako Pure Chemical, Osaka, Japan) containing penicillin, streptomycin and 10% fetal bovine serum (Equitech-Bio, Kerrville, TX). The docetaxel-resistant 22Rv1 cell line, 22Rv1DR, was previously described^18^. All cell culture experiments were performed using cells within less than 20 passages except for PC-3PRF cells stably transfected with Tet-on tetracycline-inducible perforin expression vector and docetaxel-resistant 22Rv1DR cells.

### Chemicals

Docetaxel was purchased from Selleckchem (Houston, TX, USA).

### Plasmids and transfection

The perforin expression vector for Tet-On system (pT-Rex-DEST30-perforin) was purchased from Thermo Fisher Scientific (Waltham, MA, USA). The pcDNA6/TR regulatory vector (Thermo Fisher Scientific) and pT-Rex-DEST30-perforin vector were transfected to PC-3 cells using Lipofectamine 2000 (Thermo Fisher Scientific). Transfected cells were selected under 500 μg/ml G418 and 10 μg/ml Blasticidin (Thermo Fisher Scientific). Perforin was induced by 1 μg/ml of tetracycline. The human PSA promoter-driven perforin expression vector (pDRIVEperforin-psa-hpsa) was purchased from InvivoGen (San Diego, CA, USA).

### Western blot analysis

Whole cell lysates were harvested and lysed in RIPA buffer containing the protease inhibitor cocktail (Sigma-Aldrich St. Louis, MO, USA). Western blot analysis was performed as described previously^19^. The anti-PSA and anti-β-actin antibodies were purchased from Cell Signaling Technology (Danvers, MA, USA). The immunoreactive proteins were detected using horseradish peroxidase-conjugated anti-rabbit antibody (Cell Signaling Technology) and ImmunoStar (FUJIFILM Wako Pure Chemical).

### Enzyme-Linked Immunosorbent Assay (ELISA)

Perforin expression in conditioned medium, mouse serum and harvested xenograft tumors was measured by human perforin ELISA kit according to manufacturer’s instruction (Abcam, Cambridge, UK).

### Liposome construction

SS-cleavable and pH-activated lipid-like material (ssPalmM), 1,2-Dioleoyl-sn-glycero-3-phosphoethanolamine (DOPE), cholesterol and 1,2-Dimyristoyl-rac-glycero-3-methoxypolyethylene glycol-2000 (DMG-PEG2000) were purchased from NOF Corporation (Tokyo, Japan). Encapsulation of plasmid vector in lipid nano-particles was conducted according to a previous report^20^. First, plasmid DNA and protamine solutions (0.3 mg/mL and 0.144 mg/mL) were prepared in 10 mM HEPES buffer (pH5.3). Plasmid DNA/protamine core particle was prepared by the drop-wise addition of 1 mL of the protamine solution into the 1 mL of the DNA solution with vortexing. Liposome was composed of ssPalmM, DOPE, cholesterol and DMG-PEG2000 in a molar ratio of 3:4:3:0.5. Lipids (3.3 μmol of total lipids) were dissolved in 2 mL of ethanol and the lipid solution (2 mL) was rapidly diluted with an equal volume of the plasmid DNA/protamine core particle suspension with vortexing. The solution was further diluted with 36 mL of 10 mM HEPES (pH 5.3) to obtain 5% ethanol　(v/v) concentration. The diluted solutions were concentrated to ten times by Amicon 8400 ultrafiltration stirred cell with a Biomax membrane (Merck Millipore, Allen, TX, USA) following further serial ultrafiltration with 100 mM HEPES (pH 7.4) and 10 mM HEPES (pH 7.4) using Amicon 8050 ultrafiltration stirred cell with a Biomax membrane. Finally, the liposome solution was filtrated by 0.45 μm of pore size Millex HV (Merck Millipore).

Liposome concentration was measured as total cholesterol concentration in the presence of sodium dodecyl sulfate using a Cholesterol E test Wako (FUJIFILM Wako Pure Chemical) and the total amount of fatty acids was calculated based of the molar ratio of each lipid. DNA concentration in liposomes was determined using Quant-iT Picogreen dsDNA Assay Kit (Thermo Fisher Scientific) in the presence of Triton X-100. Particle size and ς-potential were measured at 25 °C using Zetasizer Nano-S90 (Malvern Panalytical, Worcestershire, UK) after 50 times dilution of samples with distilled water.

### Human PBMCs isolation

This study was approved by the Medical Review Board of Gifu University, Graduate School of Medicine (No. 2018–219). A written informed consent was obtained from participants and blood was collected from male volunteers without clinically detectable cancer. All methods were performed in accordance with the relevant guidelines and regulations in compliance with the Declaration of Helsinki. Human PBMCs were isolated by Ficoll-Paque density gradient centrifugation according to the manufacturer’s instructions (Amersham Biosciences, Piscataway, NJ).

### Cell viability assay

Cells were seeded on 96-well plates. Twenty-four h after seeding, agents with or without PBMCs were added. Cell viability was determined using WST-1 assay kit (Roche Diagnostics, Mannheim, Germany). The mean value obtained from PBMCs alone was deducted from the values obtained from co-culture of prostate cancer cells and PBMCs.

### Xenograft model

All animal experiments were approved by the Gifu University Animal Experiment Approval Committee (No. 2019–116) and carried out in accordance with the approved guidelines. This study is compliant with the ARRIVE guidelines. Six-week-old male athymic nude mice (BALB/cSlc-nu/nu) were purchased from Japan SLC, Inc. (Shizuoka, Japan). A suspension of 22Rv1DR cells (1 × 10^7^) cells in PBS was mixed with Matrigel (1:1) in a final volume of 0.2 mL. The mixture was subcutaneously injected to generate tumors. Two weeks after the injection, tumor volume was measured and mice were randomly assigned to 2 groups (n = 5). Agents were intravenously administrated via tale vein. The tumor volume and body weight were monitored and measured once a week. Four weeks after treatment, mice were sacrificed and the resected tumors were weighed.

### Statistical analysis

Statistical analysis was performed using Graph Pad Prism 7 version 7.03 (Graph Pad Software, CA, USA). Comparison of 2 groups was made using t-test or Mann–Whitney U test. Comparison among 4 groups was made using one-way ANOVA with Tukey’s post hoc for multiple comparisons. Differences were considered significant if p < 0.05.

## Results

### Expression of perforin inhibited PC-3 cell growth

**Figure 1:**
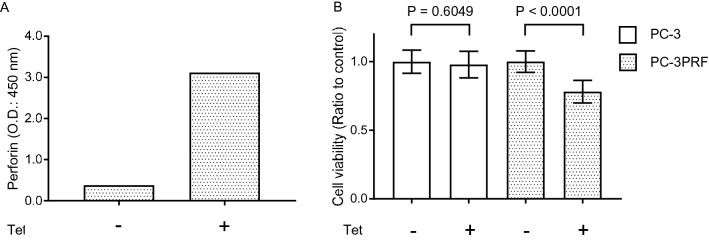
Effect of perforin induction on growth of PC-3PRF cells. (**A)** PC-3PRF cells in which perforin expression was controlled by Tet-On system were treated with tetracycline (1 μg/ml). Seventy-two h after treatment, the perforin level in conditioned medium was measured by ELISA. (**B)** PC-3PRF cells and their parental PC-3 cells were treated with tetracycline (1 μg/ml). Six days after treatment, cell viability was determined by WST-1 assay. Data shown are mean with SD (n = 10 in each group). Statistical significance was calculated using t-test.

To investigate whether perforin could exert an anti-tumor effect, we established PC-3 cell line in which perforin expression was controlled by Tet-On system (PC-3PRF). Tetracycline treatment resulted in a robust increase in extracellular perforin expression (Fig. 1A) and a significant growth inhibition of PC-3PRF cells, but did not affect the growth of parental PC-3 cells (Fig. 1B).

### Characteristics of docetaxel-resistant 22Rv1 cells

**Figure 2:**
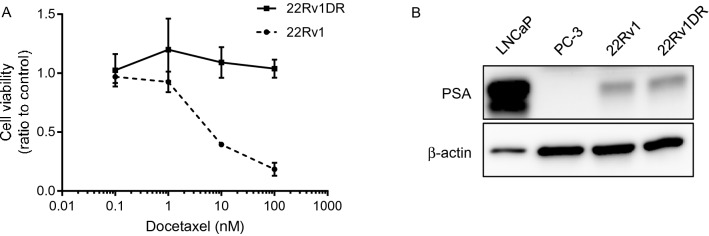
Characteristics of Docetaxel-resistant cell line, 22Rv1DR. (**A**) 22Rv1DR cells were treated with docetaxel at indicated concentrations. Seven days after treatment, cell viability was determined by WST-1 assay. Data shown are mean with SD (n = 5 in each group). (**B**) Cell lysates were harvested from LNCaP, PC-3, 22Rv1, 22Rv1DR cells and subjected to Western blot analysis for PSA and β-actin. The blots were cropped and full length blots are presented in Supplementary Information.

Advanced PC is mainly treated by androgen deprivation therapy (ADT), however, most of patients develop CRPC. CRPC is treated with chemotherapy, second generation androgen receptor inhibitor and Poly (ADP-ribose) polymerase inhibitor, but with limited success. To recapitulate clinical manifestations of PC with treatment failure for ADT and chemotherapy, we used docetaxel (DOC)-resistant CRPC cells, 22Rv1DR, which were previously established from CRPC cells, 22Rv1. Prior to the experiments, we confirmed the resistance to DOC of 22Rv1DR cells (Fig. 2A) and PSA expression in 22Rv1 and 22Rv1DR cells (Fig. 2B). As expected, PSA was absent in PC-3 cells and abundantly expressed in androgen-dependent PC LNCaP cells.

### Construction of liposomes encapsulating PSA promoter-driven perforin expression vector

**Figure 3:**
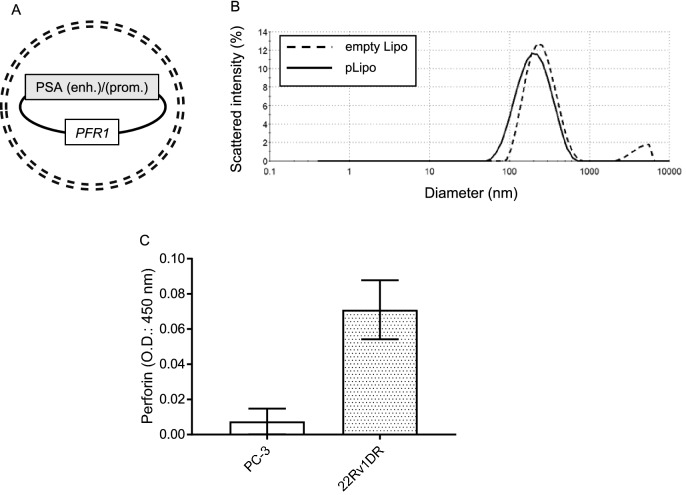
Characteristics of liposomes encapsulating PSA promoter-driven perforin expression vector. (**A**) A schema of liposomes encapsulating PSA promoter-driven perforin expression vector (pLipo). (**B**) The diameter of empty Lipo and pLipo were measured using a particle size analyzer. (**C**) PC-3 and 22Rv1DR cells (2.0 × 10^4^) were seeded on a 96-well plate and pLipo containing 0.5 μg of perforin expression vector were added to each well. Forty-eight h after treatment, the perforin expression level in conditioned medium was measured by ELISA. Data shown are mean with SD (n = 3 in each group).

The conceptual scheme of liposomes encapsulating PSA promoter-driven perforin expression vector (pLipo) is shown in Fig. 3A. The diameter (Fig. 3B), ς potential and plasmid DNA concentration of empty liposomes and pLipo were 249.0 and 153.9 nm, − 2.0 and − 14.3 mV and 0 and 25.2 μg/ml, respectively. After transfection with pLipo, perforin was detected in conditioned medium of 22Rv1DR cells expressing PSA, but not in that of PC-3 cells lacking PSA expression (Fig. 3C).

### Liposomes encapsulating perforin expression vector inhibited PC cell growth in the presence of human PBMCs

**Figure 4:**
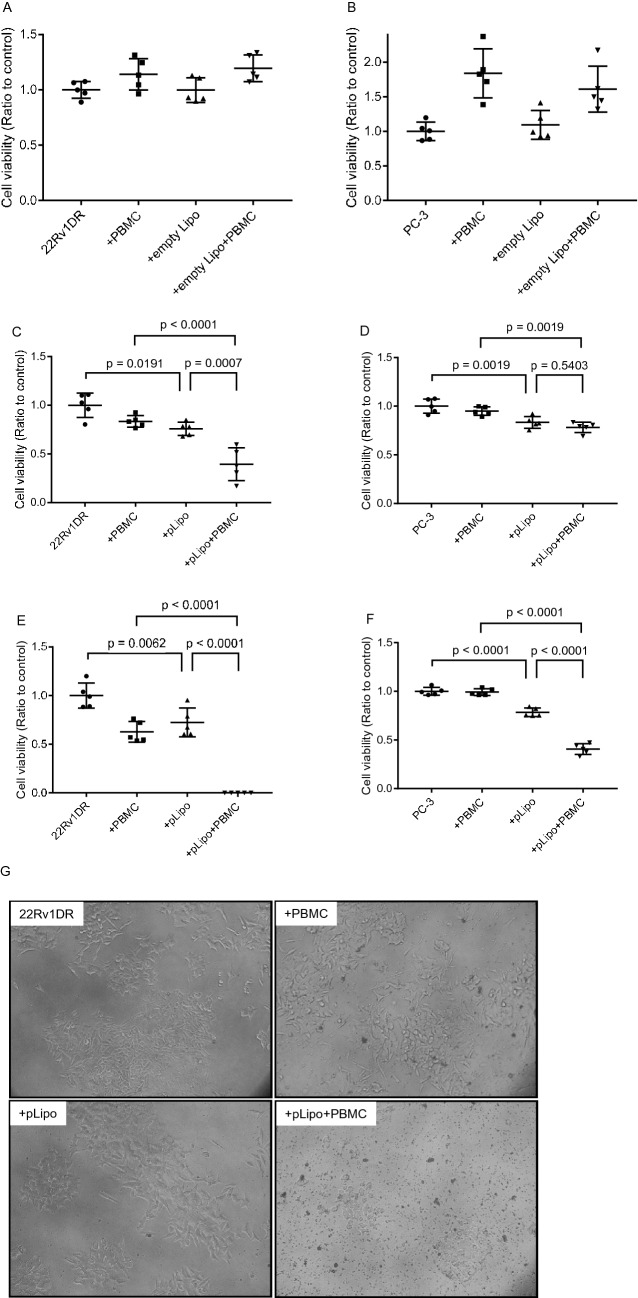
Anti-tumor effect of liposomes encapsulating PSA promoter-driven perforin expression vector in vitro. 22Rv1DR and PC-3 cells (1.0 × 10^3^) cells were seeded on 96-well plates. (**A**) and (**B**) Twenty-four h later, empty Lipo (1.89 μg of lipid/well), human PBMCs (2.0 × 10^4^) or both were added. (**C**) and (**D**) Twenty-four h later, pLipo at a low concentration (5.0 ng of perforin expression vector/well), human PBMCs (2.0 × 10^4^) or both were added. (**E**) and (**F**) Twenty-four h later, pLipo at a high concentration (169.0 ng of perforin expression vector/well), human PBMCs (2.0 × 10^4^) or both were added. Cell viability was determined by WST-1 assay 3 to 7 days after treatment. Data shown are mean with SD (n = 5 in each group). Statistical significance was calculated using one-way ANOVA with Turkey’s multiple comparison test. (G) White light microscopic Image of cells treated with pLipo at a high concentration with or without PBMCs were taken 7 days after treatment.

Since perforin functions in concert with granzymes secreted from CTLs and NK cells, the anti-tumor effect of pLipo was also evaluated in the presence of human PBMCs. First, we examined whether empty liposomes (empty Lipo) affect growth of PC cells. Either empty Lipo alone, PBMCs alone or their combination did not inhibit growth of 22Rv1DR and PC-3 cells (Fig. 4A and B, respectively). Then, we examined the anti-tumor effect of pLipo on PC cells. Treatment with pLipo at a low concentration was prone to inhibit growth of 22Rv1DR and PC-3 cells (Fig. 4C and D, respectively). The co-incubation with PBMCs, significantly enhanced growth inhibition of 22Rv1DR cells compared to pLipo alone but not of PC-3 cells. When co-incubated with PBMCs, treatment with pLipo at a high concentration resulted in nearly complete growth inhibition of 22Rv1DR cells (Fig. 4E and G), although a moderate inhibition of PC-3 cell growth was also observed (Fig. 4F).

### Liposomes encapsulating perforin expression vector decreased PC growth in vivo

**Figure 5:**
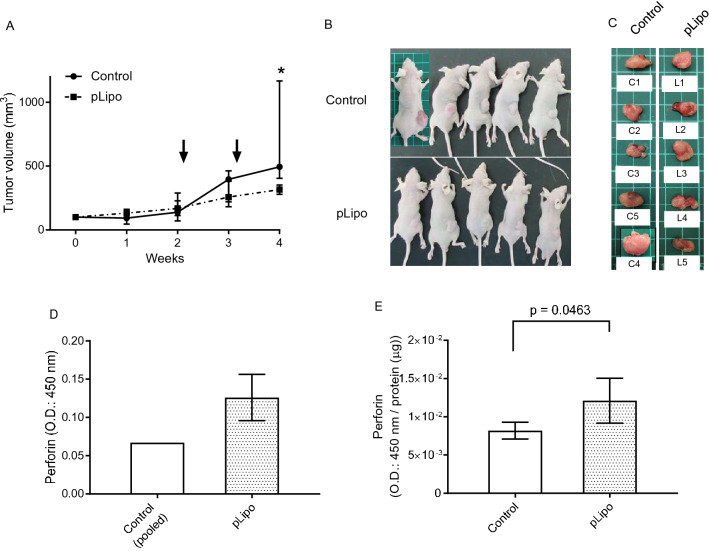
Anti-tumor effect of liposomes encapsulating PSA promoter-driven perforin expression vector in vivo. (**A**) pLipo were administrated to male nude mice bearing 22Rv1DR-derived tumor xenograft via tail vein (control: PBS, pLipo: 50.4 μg of perforin expression vector/injection). Tumor volume was measured once a week. Arrow heads indicate time when agents were injected. (**B**) Images of mice just after sacrifice. One mouse in control group was sacrificed 2 days before the planned date due to ethical reason of having developed a huge tumor. Data shown are median with range from five mice in each time point. Statistical significance was calculated using Mann–Whitney U test. *p = 0.0079 vs control at week 4. (**C**) All harvested tumors were imaged. (**D**) and (**E**) Perforin expression in serum (**D**) and lysed tumor (**E**) was measured by ELISA 24 h after injection of pLipo. The amount of intratumor perforin expression was standardized by the concentration of the lysed protein.

To investigate the anti-tumor effect in vivo, pLipo were intravenously injected to male nude mice bearing 22Rv1DR-derived tumor xenograft via tail vein. A large amount of pLipo was administered compared to that used in vitro experiments. Two weeks after subcutaneous injection of 22Rv1DR cells, tumor volume was measured and mice were randomly divided into control and pLipo groups, to which PBS or pLipo were injected at week 2 and 3, respectively. At week 4, mice were sacrificed to obtain tumor xenograft, serum, kidney and liver. As shown in Fig. 5A–C, the tumor volume in the pLipo group was significantly decreased compared to control group at week 4. The median of the calculated tumor volumes in control and pLipo groups at week 4 were 494.8 and 316.3 mm^3^, respectively. The perforin expression level measured by ELISA was elevated in both serum and tumor 24 h after injection of pLipo (Fig. 5D and E, respectively). Perforin expression in kidney and liver cannot be determined due to high background (data not shown).

## Discussion

Perforin and granzymes secreted from CTLs and NK cells play critical roles in cancer immunosurveillance. Dysfunction of perforin is demonstrated to be associated with the onset and progression of hematological cancer^8,9,21^. In PC, the intensity and frequency of perforin expression in tumor infiltrating lymphocytes were significantly decreased compared to benign prostate hyperplasia (BPH), whereas those in peripheral blood lymphocytes showed no decrement in PC^11^. In colorectal cancer, the number of CD8+ and CD16+ lymphocytes expressing perforin was decreased, which correlated with cancer progression^12^. These findings strongly support the importance of perforin in cancer immunotherapy as well as cancer immunosurveillance, but therapies targeting perforin has not yet been established. So far, there has been a report showing that direct injection of glucocorticoid-responsive perforin expression vector into xenografted lung tumors and subsequent administration of dexamethasone suppressed their growth^16^. Since advanced PC is associated with multiple metastasis, systemic delivery of perforin expression vector is preferred.

In the present study, we aimed to increase the concentration of perforin in the microenvironment of PC in order to enhance the anti-tumor immune response mediated by CTLs and NK cells. To achieve this goal, we employed a strategy to have PC cells to express perforin and release it into the microenvironment, since overexpression of perforin in immune cells in vivo is highly likely to cause systemic adverse events. A previous report showed that co-expression of perforin and granzyme B in laryngeal cancer cells exerted a potent anti-tumor activity in vitro and in vivo^22^, which supports our idea of expressing perforin in cancer cells. Effective delivery of a perforin expression vector is a prerequisite to achieve its overexpression in PC cells. Since naked therapeutic genes are rapidly degraded by nucleases^23^, we used perforin expression vector encapsulated in liposomes but not the vector alone.

Perforin expression should be strictly controlled or confined to PC cells, because of its potential cytotoxicity to normal cells. When liposomes containing perforin expression vector driven by constitutively active promoter are systemically administered, they will be taken up by all types of cells and adverse events will be inevitable. In order to minimize perforin expression in normal cells, we generated perforin expression vector driven by PSA promoter, which has been commonly used for PC-specific expression^24^. Although CRPC is developed under androgen deprivation therapy, androgen signaling axis is still one of the major therapeutic targets for CRPC^25,26^. Thus, PSA promoter is likely to be active even in CRPC.

First of all, we examined whether perforin expression in PC cells could inhibit cell growth using PC-3PRF cells. The inhibitory effect of perforin induced and released from cells on growth was relatively small. This is not surprising, because perforin is only capable of making pores on the membranes of PC cells and granzymes that cleave DNA were absent in the experimental system. In order to provide CTLs and NK cells that secrete granzymes and thereby mimic the tumor microenvironment of patients, cell viability assay was performed in the presence of human PBMCs. The co-incubation with PBMCs enhanced growth inhibition of 22Rv1DR cells treated with pLipo at a low concentration and completely inhibited growth of those treated at a high concentration. Since CTLs and NK cells are known to kill target cells by inducing apoptosis, it is likely that the anti-tumor effect observed in vivo is mediated through apoptosis as a consequence of the increased perforin level in tumor microenvironment, which remains to be confirmed by future studies. The growth of PC-3 cells was also inhibited by the co-culture of PBMCs and pLipo at a high concentration, which may be due to the leaky expression of perforin from the PSA promoter. In addition, it is known that the CpG motif in plasmid vector activates the immune system^27^, therefore the anti-tumor effect of co-incubated pLipo and PBMCs may in part be mediated by lymphocytes activated by the CpG motif on vectors delivered from pLipo. Nevertheless, we demonstrated the anti-tumor effect of perforin alone (Fig. 1B), and a stronger anti-tumor effect of pLipo and PBMCs on 22Rv1DR cells than on PC-3 cells (Fig. 4C, D, E and F), supporting the contribution of perforin to anti-tumor activity. Further experiments using liposomes containing plasmid vector without PRF will be needed to evaluate the potential role of the anti-tumor activity mediated by the CpG motif.

The administration of pLipo in vivo decreased the volume of 22Rv1DR-derived tumor xenograft with statistical difference at week 4, however the difference was not so large as in vitro studies. This might be in part because, unlike human PBMCs, T lymphocytes are deficient in immunologically compromised BALB/cSlc-nu/nu mice used as a tumor xenograft model in the present study. The mean tumor weight of the pLipo group tended to be smaller than that of control group (data not shown). However, some tumors contained necrotic tissues and were lost during harvest, making comparison difficult between two groups. Similarly to the intratumor levels, the serum levels of perforin in pLipo group was higher compared to control group, which may be explained by the presence of perforin in blood that was primarily secreted to extracellular spaces from pLipo-transfected 22Rv1DR cells in the xenografted tumor. Unlike immunologically compromised BALB/cSlc-nu/nu mice, in patients with potentially normal immunological function, increased perforin expression in tumor microenvironment is expected to enhance the anti-tumor activity of NK cells and CTLs. To mimic the clinical condition in patients, intratumor or systemic administration of PBMCs to mouse xenograft model will be required, which will disclose the precise mechanisms underlying the anti-tumor effect of perforin in tumor microenvironment. As for adverse events, no weight loss nor unusual event was observed in pLipo group after their injection (data not shown).

Similarly to PSA, prostate-specific membrane antigen (PSMA) is predominantly expressed in PC cells and thus has been used as diagnostic marker and therapeutic target in clinical setting^28^. In an effort to further ensure PC cell-specific expression of perforin, we constructed anti-PSMA antibody-conjugated pLipo for their specific delivery to PC cells. The anti-tumor effect of anti-PSMA antibody-conjugated pLipo was similar to that of pLipo in vitro and in vivo (data not shown). One possible explanation for the lack of difference is that the amount of anti-PSMA antibody conjugated to liposomes was much lower than anticipated, which remains to be solved in the future.

In summary, we demonstrated that liposome-mediated PC-specific expression of perforin could be a novel therapy for advanced PC patients.

## Supplementary Information

Supplementary Information.
