# Supplementary material for: Gene therapy of prostate cancer using liposomes containing perforin expression vector driven by the promoter of prostate-specific antigen gene
Source: Sci Rep. 2022 Jan 27;12:1442. doi: 10.1038/s41598-021-03324-6 (PMC8795355; doi:10.1038/s41598-021-03324-6)
Supplement: Supplementary file 1 — Supplementary Information. [file 41598_2021_3324_MOESM1_ESM.pptx]

## Slide 1
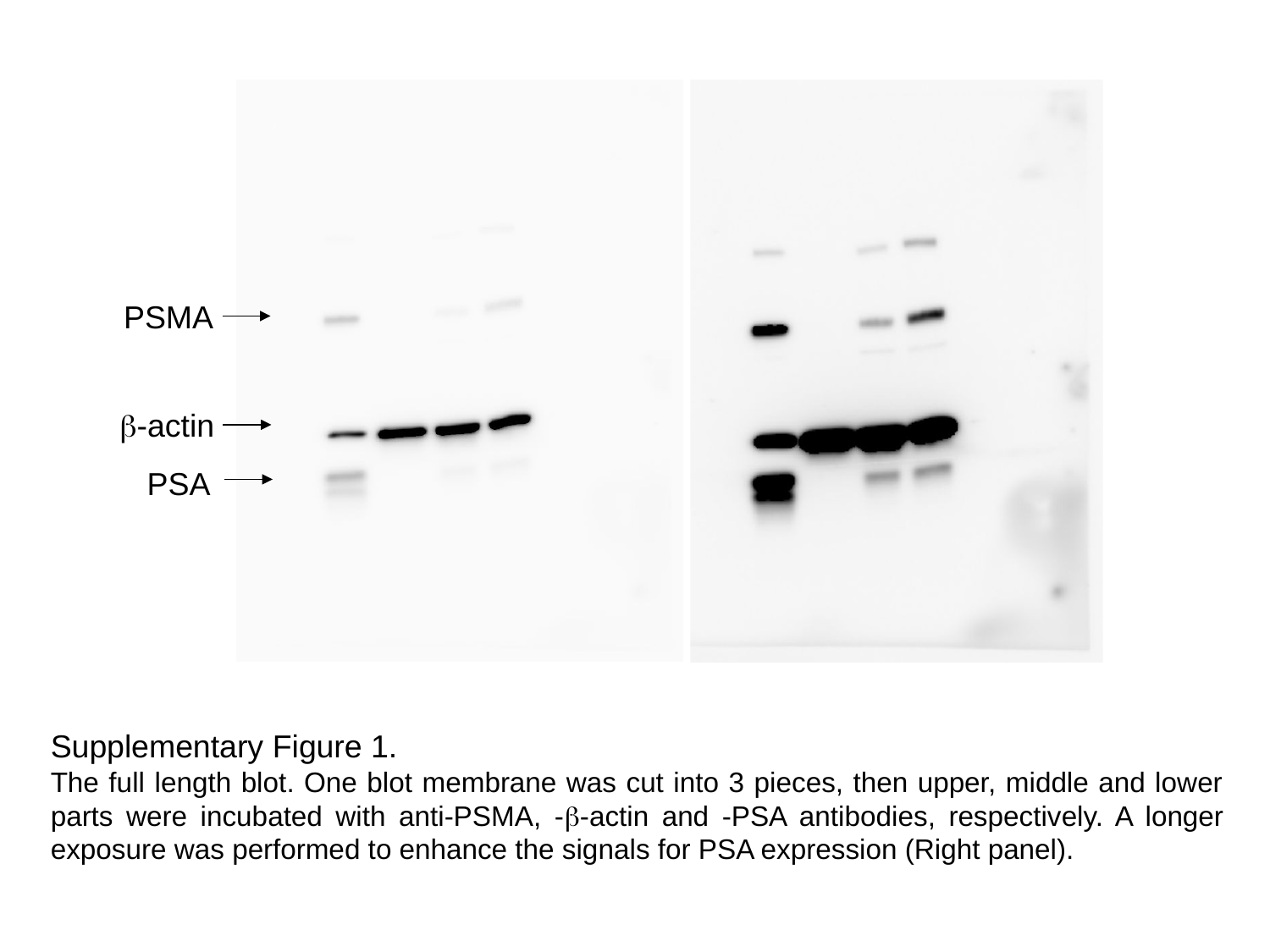

PSMA
b-actin
PSA
Supplementary Figure 1.
The full length blot. One blot membrane was cut into 3 pieces, then upper, middle and lower parts were incubated with anti-PSMA, -b-actin and -PSA antibodies, respectively. A longer exposure was performed to enhance the signals for PSA expression (Right panel).
